# Clinical and molecular heterogeneity in advanced-stage *POLE*-mutated endometrial carcinoma: a focus on aggressive subsets and co-mutations

**DOI:** 10.3389/fonc.2026.1800797

**Published:** 2026-04-29

**Authors:** Nicolo Cavasin, Sami Kilzie, Mattia Vinci, Rita Trozzi, Camilla Nero, Stefano Restaino, Chiara Cassani, Sandro Pignata, Carmela Pisano, Giuseppe Scibilia, Robert Fruscio, Rosanna Mancari, Adolfo Favaretto, Matteo Fassan, Luisa Toffolatti, Giovanna Gallina, Francesco Fanfani, Vanda Salutari, Grazia Artioli

**Affiliations:** 1 Department of Medical Oncology, Azienda Unità Locale Socio-Sanitaria 2 Marca Trevigiana, Treviso, Italy; 2 Department of Obstetrics and Gynecology, Ospedale di Treviso, Treviso, Italy; 3 Department of Pathology, Treviso General Hospital, Treviso, Italy; 4 Department of Women’s, Children’s health, Fondazione Policlinico Universitario A. Gemelli, Istituto di Ricovero e Cura a Carattere Scientifico, Rome, Italy; 5 Catholic University of the Sacred Heart, Rome, Italy; 6 Clinic of Obstetrics and Gynecology, Santa Maria della Misericordia University Hospital, Azienda Sanitaria Universitaria Friuli Centrale, Udine, Italy; 7 Department of Clinical, Surgical, Diagnostic, and Pediatric Sciences, University of Pavia, Unit of Obstetrics and Gynecology, Istituto di Ricovero e Cura a Carattere Scientifico (IRCCS) San Matteo Foundation, Pavia, Italy; 8 Department of Urology and Gynecology, Istituto Nazionale Tumori Istituto di Ricovero e Cura a Carattere Scientifico Fondazione G. Pascale, Naples, Italy; 9 Gynecology Unit, Giovanni Paolo II Hospital, Ragusa, Italy; 10 Unità Operativa (Operative Unit) Gynecology, Department of Medicine and Surgery, University of Milan-Bicocca, Istituto di Ricovero e Cura a Carattere Scientifico San Gerardo dei Tintori, Monza, Italy; 11 Gynecologic Oncology Unit, Department of Experimental Clinical Oncology, Istituto di Ricovero e Cura a Carattere Scientifico (IRCCS) Regina Elena National Cancer Institute, Rome, Italy; 12 Veneto Institute of Oncology, Istituto di Ricovero e Cura a Carattere Scientifico (IOV-IRCCS), Padua, Italy

**Keywords:** Advanced-stage cancer, endometrial cancer, molecular factors, mutation - genetics, POLE mutation (POLE mut)

## Abstract

**Background:**

The POLE-mutated molecular subtype of endometrial carcinoma is characterized by an ultramutated genomic profile, high tumor mutational burden, and a favorable prognosis. The clinical behavior of advanced-stage POLE-mutated endometrial carcinoma remains poorly defined due to its rarity. While most cases retain an indolent course, emerging evidence suggests that a subset may develop aggressive disease, including atypical metastatic patterns such as brain dissemination.

**Methods:**

A multi-center retrospective analysis was performed on 14 patients with advanced (International Federation of Gynecology and Obstetrics Stage III-IV) POLE-mutated endometrial carcinoma. Data on the clinico-pathological characteristics and treatment outcomes were collected.

**Results:**

The cohort consisted exclusively of high-grade tumors (100% Grade 3), with the majority being endometrioid (86.7%). Lymphovascular space invasion was ubiquitous (100%). The detected POLE mutations were: p.V411L (37%), p.P286A (14%), p.S459F (14%), p.G433A (7%), p.M444L (7%), p.S297P (7%), p.P286R (14%). Most tumors were mismatch repair-proficient (64%) and p53 wild-type (79%). Co-mutations in the PI3K/AKT pathway were detected in 29% of cases, while 43% of patients harbored at least one additional oncogenic driver. Data were interpreted according to variant allele frequency, in order to differentiate biologically relevant clonal alterations from subclonal or passenger events. Three patients (21%) underwent surgery followed by adjuvant radiotherapy and chemotherapy, three (21%) received surgery followed by chemotherapy alone, and four (29%) were treated with surgery alone; treatment data were unavailable for one patient (7%). One patient (7%) presented with brain metastases at diagnosis.

**Conclusion:**

This study provides a detailed descriptive characterization of a rare endometrial carcinoma patient population and highlights the emergence of atypical metastatic patterns, including cerebral involvement. Although observed in a very limited number of cases, the recurrent identification of the V411L variant among patients with advanced disease raises hypotheses and requires validation in larger cohorts. These findings underscore the need for nuanced risk stratification that goes beyond POLE mutation status alone.

## Introduction

The molecular classification of endometrial carcinoma (EC) has profoundly refined risk stratification and clinical management (1). Among the four molecular subgroups defined by The Cancer Genome Atlas (TCGA), *POLE*-mutated tumors represent a distinct entity characterized by pathogenic exonuclease domain mutations of the polymerase epsilon gene. These cancers are typically high-grade, endometrioid, and exhibit an ultramutated phenotype with a very high tumor mutational burden (TMB), resulting in robust anti-tumor immune responses and an exceptionally favorable prognosis (1–3). This prognostic advantage persists even in the presence of otherwise prognostically unfavorable pathological features such as substantial lymphovascular space invasion (LVSI), p53 aberrant, or non-endometrioid histology (3). This strong survival signal has prompted a paradigm shift in clinical practice, with recent international guidelines recommending adjuvant therapy de-escalation in early-stage disease and selective omission even in advanced stages (4). Nonetheless, the evidence underpinning these recommendations is derived almost exclusively from early-stage cohorts, and little is known about the clinical trajectory of advanced-stage disease (FIGO III–IV) (2, 5). Although recurrences remain uncommon, case reports and small series have documented atypical metastatic patterns, including cerebral dissemination, challenging the assumption of uniformly indolent behavior (3, 6). The mechanisms underlying this heterogeneity are incompletely understood. Potential contributors include the type of *POLE* mutation itself, the interplay with the tumor immune microenvironment, and the acquisition of co-operating oncogenic drivers such as PI3K/AKT pathway alterations (7). Against this background, systematic characterization of advanced-stage *POLE*-mutated EC is urgently needed to clarify its biological diversity, to identify subsets at risk of aggressive disease, and ultimately to guide tailored therapeutic strategies.

## Methods

### Study population and data collection

This analysis was conducted within the framework of the MITO POLE END multicenter retrospective study, approved by the Institutional Review Boards of all participating centers. Participating centers retrospectively identified patients with endometrial carcinoma harboring a pathogenic mutation in the *POLE* exonuclease domain. For the present group analysis, only patients with advanced-stage disease (FIGO 2023 stages III–IV) were included. All patients had provided written informed consent for the use of clinical and molecular data for research purposes, in accordance with local institutional requirements. The study involved 20 Italian oncology and gynecologic oncology centers. The study protocol was approved by the ethics committees of all participating institutions and was conducted in accordance with the principles of the Declaration of Helsinki.

### Clinicopathological data collection

Extracted variables included age at diagnosis, histological subtype, tumor grade, FIGO 2023 stage, presence of LVSI, immunohistochemical profile (MMR proteins, p53, estrogen receptor [ER], progesterone receptor [PR]), detailed *POLE* mutation characteristics, co-occurring somatic mutations, administered treatments (surgery, systemic therapy, radiotherapy), and oncologic outcomes.

### Molecular analysis

*POLE* mutational status was assessed locally at each participating center using next-generation sequencing (NGS)–based assays. Variant pathogenicity was assessed using major public databases (ClinVar, COSMIC, OncoKB) in accordance with AMP/ASCO/CAP recommendations (8) Immunohistochemistry was used to assess MMR and p53 status in accordance with ESGO/ESTRO/ESP and ESMO guideline recommendations. No central pathology or molecular review was performed. Given the ultramutated nature of *POLE*-mutated tumors, co-mutation analyses were interpreted with particular attention to variant allele frequency (VAF), known oncogenic relevance, and consistency with prior literature.

### Statistical analysis

Descriptive statistics were used to summarize the cohort’s characteristics. Continuous variables were reported as medians with ranges, and categorical variables as frequencies and percentages.

## Results

### Clinico-pathological features

Fourteen patients with advanced-stage *POLE*-mutated endometrial cancer were included. Patients were diagnosed and treated between 2021 to 2024. The median age at diagnosis was 58 years (range 32–86). All tumors were FIGO Grade 3. Endometrioid histology was observed in 12 cases (86%), while 2 tumors (14%) showed a non-endometrioid histology (one clear cell and one undifferentiated/dedifferentiated carcinoma). FIGO stage III disease was observed in 11 patients (79%), whereas stage IV disease was documented in 3 patients (21%). Among the 14 patients, three (21%) underwent surgery followed by adjuvant radiotherapy and chemotherapy, three (21%) received surgery followed by chemotherapy alone, and six (43%) were treated with surgery alone, one received surgery followed by radiotherapy (7%); treatment data were unavailable for one patient (7%) **Table 1**.

**Table 1:** Characteristics of advanced-stage POLE-mutated endometrial carcinoma patients (n=14).

Characteristic	N 14 (%)
Age at Diagnosis	58.6 (32-86)
Histology	
Endometrioid Serous undifferentiated histology	12 (86%) 1 (7%) 1 (7%)
FIGO Stage III IV	11 (79%) 3 (21%)
LVSI Present	14 (100%)
Treatment surgery+ radiotherapy+ chemotherapy surgery + chemotherapy surgery surgery + radiotherapy treatment unavailable	3 (21%) 3 (21%) 6 (43%) 1 (7%) 1 (7%)
*POLE* mutation V411L P286R S459F P286R S297F M444K G433R	5 (37%) 2 (14%) 2 (14%) 2 (14%) 1 (7%) 1 (7%) 1(7%)
MMR Status Proficient (pMMR) Deficient (dMMR) Not Determined	9 (64%) 4 (29%) 1 (7%)
p53 Status Wild-Type Mutant	11 (79%) 3 (21%)
Co-mutations Any *PI3K/AKT* *PTEN* Other No data	4 (29%) 2 (14%) 6 (43%) 2 (14%)
Recurrence	1 (7%)
Brain metastases	1 (7%)

### Molecular profile

The molecular landscape of the study cohort was dominated by hotspot mutations in the *POLE* exonuclease domain. The most frequent variant was p.Val411Leu (V411L), identified in 37% of cases (n=5), followed by p.Pro286Arg (P286R) and p.Ser459Phe (S459F), each detected in 14% of tumors (n=2 each).

Additional, less frequent non-hotspot *POLE* exonuclease-domain variants included p.Gly433Arg, p.Met444Lys, and p.Ser297Phe; although less common, these variants have been previously reported in endometrial carcinoma, with p.Met444Lys and p.Ser297Phe included among pathogenic *POLE* exonuclease-domain mutations according to the framework proposed by León-Castillo et al. (9) With regard to other molecular classifiers, aberrant p53 expression was observed in three tumors, one of which also showed loss of MSH2 and MSH6 expression, consistent with MMR deficiency. Among the remaining eleven tumors with wild-type p53, seven were classified as MMR proficient, while the others showed MMR deficiency or could not be assessed. Overall, these findings indicate that, despite the defining role of pathogenic *POLE* mutations, 7 of the 14 tumors in our cohort exhibited overlapping molecular features and were therefore classified as multiple-classifier cases, including three tumors with concomitant aberrant p53 expression and four tumors showing loss of MSH2 expression. This supports the presence of multiple molecular classifiers within advanced *POLE*-mutated endometrial cancer.

### Outcomes

With a median follow-up of 23 months, disease recurrence was observed in 1 patient (7%), despite having received adjuvant chemotherapy in the postoperative setting. This patient developed an isolated pelvic recurrence and subsequently started immunotherapy, which is currently ongoing with clinical benefit. The tumor harbored a POLE exonuclease domain mutation (V411L) and did not meet criteria for a multiple classifier, being characterized by p53 wild-type status and proficient mismatch repair (pMMR). This observation is consistent with the well-documented high sensitivity of POLE-mutated tumors to immune checkpoint inhibition and further supports the effectiveness of immunotherapy in this molecular subset, even in the recurrent setting. Separately, one patient presented with central nervous system (CNS) involvement at diagnosis, representing a rare but clinically relevant pattern of disease presentation in this cohort. This patient also carried a concomitant *PIK3CA* mutation, raising the hypothesis of cooperative oncogenic mechanisms contributing to CNS dissemination. All patients received multimodal treatment strategies, including primary surgery followed by systemic therapies and/or radiotherapy according to disease stage, an aspect that is crucial for the interpretation of survival outcomes in this cohort. The patient with brain metastases died of disease. The majority of the patients (87%) were alive, with no evidence of disease (NED) at last contact. Overall, these findings confirm the generally favorable prognosis of *POLE*-mutated tumors.

## Discussion

Our study provides a comprehensive characterization of advanced-stage *POLE*-mutated endometrial cancer, confirming its overall favorable prognosis while highlighting relevant biological and clinical nuances. Despite the universal presence of adverse pathological features, including high-grade histology and lymphovascular space invasion, clinical outcomes remained excellent, supporting the concept that the strong immunogenicity associated with pathogenic *POLE* mutations may effectively counterbalance aggressive pathological characteristics (1, 2, 4, 5). Notably, half of the patients in our cohort were classified as multiple-classifier tumors, due to the coexistence of aberrant p53 expression or loss of MSH2 expression (10). Nevertheless, according to the established molecular hierarchy, all cases were ultimately assigned to the *POLE*-mutated group, and the presence of additional molecular alterations did not appear to translate into an adverse clinical impact (3, 7). This observation supports the hypothesis that, in *POLE*-mutated tumors, concomitant molecular alterations may play a limited prognostic role, potentially overridden by the highly immunogenic, “hot” tumor microenvironment characteristic of this molecular subtype (3, 7).During follow-up, only a single recurrence was observed at a median follow-up approaching two years. This finding is particularly relevant considering that, in endometrial cancer, the majority of recurrences are known to occur within the first three years after diagnosis (4). The low recurrence rate observed in our cohort therefore reinforces the notion of an excellent prognosis even in advanced-stage *POLE*-mutated disease. A single case of brain metastasis was observed in a patient carrying the *POLE* p.Val411Leu hotspot mutation (6). While brain metastases from endometrial cancer are exceedingly rare and generally associated with poor outcomes (11–13), emerging evidence suggests that their clinical behavior in *POLE*-mutated tumors may differ substantially from that of conventional endometrial carcinomas (6). However, given the isolated nature of this observation, no definitive conclusions regarding metastatic tropism or variant-specific behavior can be drawn, and further validation in larger cohorts is warranted. Overall, our findings indicate that advanced *POLE*-mutated endometrial cancer retains an excellent prognosis, even in the presence of multiple molecular classifiers or additional driver alterations (1–7, 10). These data further support the dominant prognostic role of pathogenic *POLE* mutations and suggest that co-occurring molecular events may have limited impact on disease behavior within this highly immunogenic subgroup.

The classification of endometrial carcinoma in cases harboring more than one molecular classifier represents a clinically relevant challenge that deserves specific consideration. According to the established molecular hierarchy, *POLE* mutations take precedence over other classifiers, including p53 aberrancy and mismatch repair deficiency (10). In practical terms, this means that a tumor simultaneously carrying a pathogenic *POLE* mutation and aberrant p53 expression — or mismatch repair deficiency — should still be assigned to the *POLE*-mutated molecular subgroup for the purposes of prognostication and treatment decision-making. In our cohort, half of the patients were classified as multiple-classifier tumors; nonetheless, their clinical outcomes remained favorable, consistent with the dominant prognostic role of pathogenic *POLE* mutations. These observations support the current guideline recommendation to assign *POLE*-mutated status as the primary classifier regardless of co-occurring molecular alterations, while acknowledging that the biological implications of concurrent molecular events in advanced-stage disease remain incompletely understood and merit further prospective investigation (3, 7, 10, 14).

## Conclusion

Advanced-stage POLE-mutated endometrial carcinoma represents a rare and biologically heterogeneous entity. Our findings confirm that pathogenic POLE mutations confer a favorable prognosis even in the presence of adverse pathological features and advanced disease stage. Nevertheless, the coexistence of additional molecular alterations — including aberrant p53 expression, mismatch repair deficiency, and PI3K/AKT pathway co-mutations — warrants careful consideration, as their prognostic impact in this setting remains to be fully elucidated. The observation of atypical metastatic patterns, such as central nervous system involvement, underscores the biological complexity of a subset of these tumors. Current evidence is insufficient to support treatment de-escalation strategies in advanced-stage disease, and prospective data from larger cohorts are urgently needed to refine risk stratification and guide adjuvant treatment decisions in this molecular subgroup.

## Data Availability

The original contributions presented in the study are included in the article/supplementary material, further inquiries can be directed to the corresponding author/s.
